# Usefulness of Faecal Calprotectin Measurement in Children with Various Types of Inflammatory Bowel Disease

**DOI:** 10.1155/2012/608249

**Published:** 2012-05-14

**Authors:** Marzena Komraus, Halina Wos, Sabina Wiecek, Maciej Kajor, Urszula Grzybowska-Chlebowczyk

**Affiliations:** ^1^Department of Paediatrics, Medical University of Silesia, Medykow 16, 40-752 Katowice, Poland; ^2^Department of Pathomorphology, Medical University of Silesia, 40-752 Katowice, Poland

## Abstract

*Introduction*. The aim of the study was to assess the usefulness of the FC measurement in children with various types of IBD and relation to the disease activity. *Patients and Methods*. 91 patients (49 boys: 53.85% and 42 girls: 46.15%, mean age: 13.38 years, range 6–18 years) were included in the analysis. Patients were divided into the groups: B1—24 children with CD, B2—16 patients with UC, and a group comprising 31 children with other types of colitis; the control group (K) comprised 20 healthy children. FC was assayed by ELISA method, using Phical test (Calpro). *Results*. The mean faecal calprotectin concentrations were higher in children with CD and UC as compared to healthy controls, patients with eosinophilic, lymphocytic, and nonspecific colitis. A positive correlation was observed between FC concentrations and the disease activity (the PCDAI scale, the Truelove-Witts Scale, and the endoscopic Rachmilewitz Index). *Conclusion*. It seems that the FC concentrations can be a useful, safe, and noninvasive test in children suspected for IBD, since FC concentration is higher in children with CD and UC than in patients with other inflammatory diseases.

## 1. Introduction

Inflammatory bowel disease (IBD) is a common diagnostic and therapeutic problem, affecting patients at increasingly young ages. A standard diagnostic method includes macroscopic assessment of the intestinal mucosa during colonoscopy and histopathological evaluation of the obtained biopsies. According to Porto criteria, the diagnosis of IBD is based on the clinical presentation, intestinal endoscopic and histological features, laboratory tests, and radiological examination.

There is a need for better, noninvasive methods facilitating the process of diagnosing. Recently, reports on the usefulness of a noninvasive examination, that is, faecal calprotectin measurement in IBD diagnosis, have been published. Calprotectin (a neutrophil protein) is present in both blood serum and faeces. Its concentration considerably increases during infections and inflammatory conditions, including IBD. Evaluation of faecal calprotectin (FC) seems to be a screening test selecting patients requiring further invasive diagnostics.

Furthermore, there have been reports on using calprotectin assays in monitoring the treatment of Crohn's disease and ulcerative colitis in adults and children [1–4].

To our knowledge, there are no published data on the usefulness of faecal calprotectin assays in the diagnosis of other atypical forms of bowel inflammation, such as eosinophilic, lymphocytic, or nonspecific colitis.

## 2. Aim of Study

The aim of the study is to assess the usefulness of faecal calprotectin measurement in children with various types of IBD and to evaluate FC concentration in children with Crohn's disease and ulcerative colitis in relation to disease activity.

## 3. Patients and Methods

91 patients were included in the analysis, including 49 boys (53.85%) and 42 girls (46.15%), ranging from 6 to 18 years of age (the mean age was 13.38 years). The study group comprised 71 children with various types of IBD, who were subsequently divided into six subgroups: B1—24 (33.8%) children with Crohn's disease (CD), B2—16 (22.5%) with ulcerative colitis (UC), B3—7 (9.8%) with eosinophilic colitis (EC), B4—8 (11.26%) with lymphocytic colitis (LC), and B5—16 (22.5%) with nonspecific colitis (NC, colitis indeterminata—CI). The control group (K) comprised 20 healthy, age- and sex-matched subjects. Patients with IBD underwent following procedures: anamnesis, physical examination, laboratory tests (inflammatory state markers, biochemical parameters of liver, pancreas, and kidney function, sweat test, coprological tests, and immunoassays), diagnostic imaging (abdominal ultrasound), and endoscopy with histopathological evaluation.

In all patients, faecal calprotectin was measured by means of ELISA method, using Phical test (Calpro). Faeces samples were obtained prior to administration of laxatives preparing patients for colonoscopy. Calprotectin concentrations ranging from 0 to 50 mg/kg were considered to be normal reference values.

Data analysis was performed with Statistica software (Microsoft). The results were evaluated by using the following tests: Kołmogorow-Smirnow, *t*-student, *U* Mann-Whitney, Fisher, and Yates; the analysis of correlation was based on the Spearman's rank correlation coefficient. A *P* value of <0.05 was considered statistically significant.

All patients and their caregivers gave informed consent to participate in the study, which was approved by the Bioethics Committee of the Medical University of Silesia in Katowice (Consent no. L.dz.NN-6501-189/05/06).

## 4. Results

A statistically significant increase in the mean concentrations of faecal calprotectin was observed in the group of children with CD and UC, as compared to the control group.

**Figure 1:**
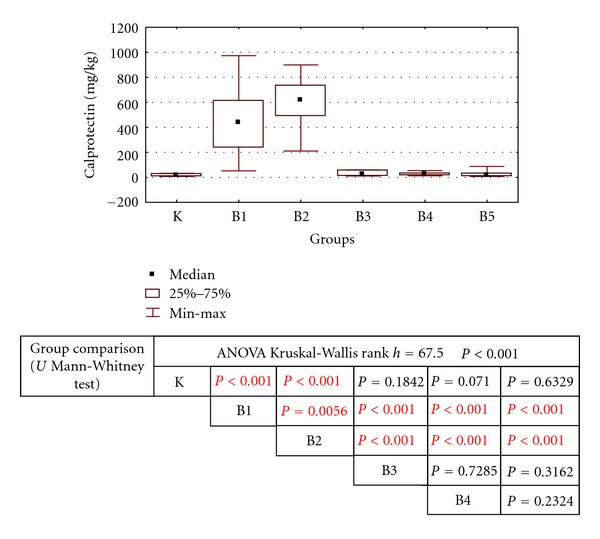
Comparison of faecal calprotectin concentrations among study groups.

Concentrations of FC were also higher in children with UC than in patients with CD. Faecal calprotectin concentrations were within the normal limits in patients with eosinophilic, lymphocytic, and nonspecific colitis, similarly to the healthy subjects (Table 1, Figure 1)

**Figure 2:**
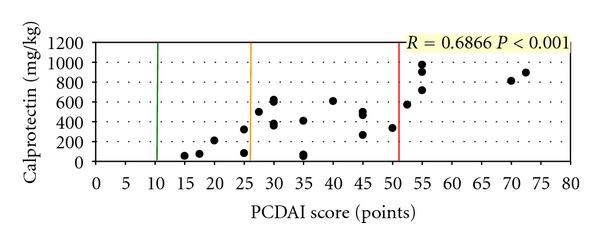
Correlation between calprotectin concentrations and the PCDAI score in CD children.

**Figure 3:**
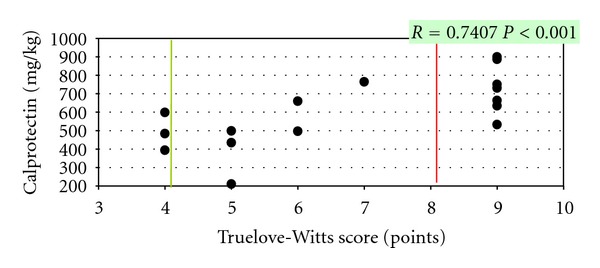
Correlation between calprotectin concentrations and the Truelove-Witts score in UC children.

**Figure 4:**
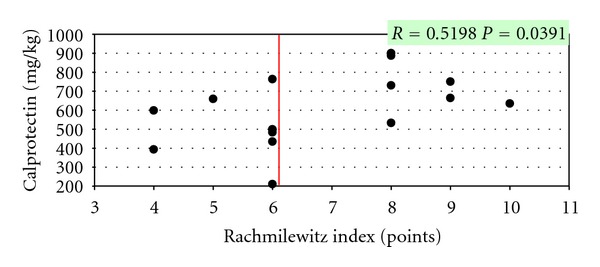
Correlation between calprotectin concentrations and the Rachmilewitz index in children with UC.

In children with CD, faecal calprotectin concentrations positively correlated with the disease severity assessed according to the PCDAI scale. In patients suffering from UC, faecal calprotectin also positively correlated with the Truelove-Witts scale and the Rachmilewitz endoscopic index (Figures 2, 3, and 4).

**Figure 5:**
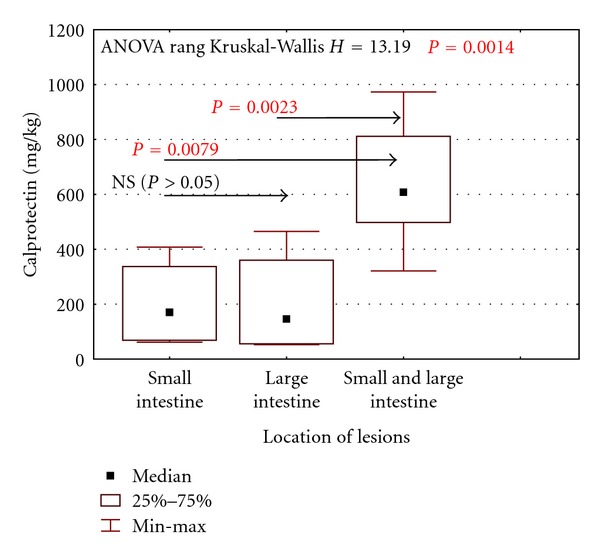
Calprotectin concentrations in relation to the location of lesions in CD patients.

A significant increase in faecal calprotectin concentrations was observed in children suffering from CD, with lesions located in both small and large intestine, and in patients presenting with inflammatory changes in 5 or more sections of the intestine (Figure 5).

## 5. Discussion

Faecal calprotectin is a promising, noninvasive screening method for diagnosing patients suffering from gastrointestinal disorders, such as abdominal pain or diarrhea, which are also typical for IBD [5–7].

So far, many authors have considered calprotectin as a useful marker in differential diagnosis of IBD and functional gastrointestinal disorders (e.g., irritable bowel syndrome) [8, 9].

Many authors evaluated faecal calprotectin concentrations in patients with suspected inflammatory process of the large intestine. Fagerberg et al. demonstrated in the group of paediatric patients that this assay is characterised by 95% sensitivity and 93% specificity, and high calprotectin concentrations show strong positive correlation with the presence of inflammatory lesions in the large intestine. According to these authors, calprotectin measurement could be a screening test preceding invasive endoscopic examinations [7].

So far, only limited studies, based on very small groups of patients, evaluated faecal calprotectin concentrations in patients with other types of bowel inflammations less common than CD and UC, such as lymphocytic, eosinophilic, and nonspecific colitis, which seem to be a considerable clinical problem in everyday pediatric practice.

In studies conducted by Bunn et al., two children with nonspecific colitis and 3 children with allergic colitis were included in the analysis. In both cases, calprotectin concentrations were found to be within the normal ranges [10].

In our study involving 31 children: 7 with eosinophilic colitis, 8 with lymphocytic colitis, and 16 with nonspecific colitis, respectively, calprotectin concentrations were also within the normal limits.

These results support the hypothesis that in the aforementioned types of bowel inflammation, histopathological examination does not reveal infiltrations of neutrophil cells, whose cytosols contain calprotectin. Therefore, its concentration in faeces is directly related to the number of neutrophils in the large intestine lumen [11, 12].

In paediatric patients, the diagnosis of nonspecific colitis (indeterminata colitis) remains unchanged in approximately 36%. Over time in some patients, the diagnosis may be changed into ulcerative colitis (in approximately 33–72.5%) or Crohn's disease (in approximately 17–27.5%) [13].

In our group of 6 CD patients, previously diagnosed conditions included single cases of ulcerative colitis, lymphocytic colitis, and nonspecific colitis, whereas eosinophilic colitis was found in 3 subjects. Increased calprotectin concentrations may be useful when making a decision on extending diagnostic procedures in patients with less frequent types of bowel inflammation.

In our study, the mean calprotectin concentration in the examined patients with IBD was higher than it was observed by Bremner et al.; however, patients in remission were also enrolled in the latter research [14].

In the presented material, a significant correlation was demonstrated between calprotectin activity and the disease severity assessed by the modified PCDAI scale for CD and the modified Truelove-Witts scale and the Rachmilewitz endoscopic index for UC.

A similar analysis was performed by Kobelska-Dubiel et al., demonstrating a strong positive correlation between calprotectin concentrations and the disease severity according to the modified Truelove-Witts scale only in UC [4].

Analyses performed in children assessed a correlation between FC concentrations and intensity of macroscopic and microscopic inflammatory lesions in the large intestine, observed in the course of IBD. Studies carried out by Fagerberg et al. included 39 children with IBD, in whom calprotectin concentrations strongly correlated with intensity and extent of micro- and macroscopic abnormalities [15].

Norwegian researchers also confirmed such a correlation and, moreover, suggested that intensity of inflammatory lesions rather than their extent influences faecal calprotectin concentration [16]. This suggestion can be supported by data obtained from our study, indicating that in patients with UC calprotectin concentration depends on disease activity and not on the extent of lesions, in contrast to patients suffering from CD. In the latter group, calprotectin concentrations correlated with the disease activity and were significantly higher in children with inflammatory lesions present in both small and large intestine and located in 5 or more sections of the large intestine. These results support conclusions from the studies on clinical expression of the disease, which demonstrated that in children and adolescents the disease is more severe and CD lesions are located in the small intestine [17, 18].

So far, none of the faeces examinations appeared useful in the routine diagnostics of IBD. Faecal calprotectin measurement seems a promising test to evaluate disease activity and a tool for monitoring IBD treatment. In everyday practice, calprotectin assay may become a screening test preceding a decision on invasive endoscopic examination. A disadvantage of this method is its low specificity, which is connected with the fact that in patients with increased calprotectin concentration many organic diseases should be excluded.

## 6. Conclusion

It seems that measurement of faecal calprotectin concentration can be a useful, safe, and noninvasive test in children suspected for IBD, since it is found to be increased in children with CD and UC as compared to patients with other inflammatory diseases (eosinophilic, lymphocytic, and nonspecific colitis) and also in the reference to healthy subjects.

When the faecal calprotectin concentration is increased in children with less common types of bowel inflammation, a further follow-up of such patients is recommended.

Faecal calprotectin concentration correlates positively with the disease severity in CD and UC patients; thus, it may be useful when choosing or modifying the appropriate treatment regimen.

## Figures and Tables

**Table 1:** Faecal calprotectin concentrations in the study subjects.

		Group K	Group B1 (CD)	Group B2 (UC)	Group B3 (EC)	Group B4 (LC)	Group B5 (NC)
		*n* = 20	*n* = 24	*n* = 16	*n* = 7	*n* = 8	*n* = 16
Faecal calprotectin [mg/kg]	*ρε* *δ*.	**22.5**	**448.1**	**601.1**	**28.5**	**17.1**	**31.4**
	SEM	2.1	57.9	45.8	7.7	2.1	6.2
	Median	14.5	436.8	616.0	24.0	12.9	26.0
	Min–Max	7.1–87.5	52.5–973.4	210.5–899.5	10.3–58.7	7.5–31.4	12.5–59.6
